# Correlation analysis of flow parameters in the olfactory cleft and olfactory function

**DOI:** 10.1038/s41598-022-25282-3

**Published:** 2022-12-02

**Authors:** Shuo Wu, Peiji Wang, Dielai Xie, Feitong Jian

**Affiliations:** 1 E.N.T. Department, The 3rd Affiliated Hospital, Sun Yat-Sen University, Guangzhou, People’s Republic of China; 2 School of Physics and Astronomy, Sun Yat-Sen University, Zhuhai, People’s Republic of China; 3 Radiology Department, The 3rd Affiliated Hospital, Sun Yat-Sen University, Guangzhou, People’s Republic of China

**Keywords:** Preclinical research, Computational models

## Abstract

The olfaction is related to flow in the olfactory cleft. However, There is a lack of studies on the relationship between flow characteristics of the olfactory cleft and olfactory function. In this study, the anatomical structure of the olfactory cleft was reconstructed in three dimensions using the raw data obtained from the CT scans of sinuses of 32 enrolled volunteers. The Sniffin’ Sticks test was used to examine the olfaction. We investigated the correlation between airflow parameters and olfactory function of the olfactory cleft in healthy adults by the computational fluid dynamics method. We found that three parameters, airflow, airflow velocity, and airflow ratio, were highly positively correlated with olfactory function. The mean pressure was not correlated with the olfactory function. Furthermore, there is the strongest correlation between air flow through the olfactory cleft and olfactory function. The correlation between the mean velocity in the anterior olfactory cleft region and olfaction was relatively poor, while the airflow velocity at the posterior olfactory cleft region was enhanced gradually. The correlation between the airflow ratio and olfaction was optimal in the initial position of superior turbinate. The flow parameters in the posterior olfactory cleft area were more stable.

## Introduction

As an essential function of the nasal cavity, the olfactory function is affected by the flow through the olfactory cleft and the unique anatomy of the nasal cavity^1,2^. Flow through the olfactory cleft is one of the necessary conditions for the production of olfaction. If vitamin B1 is injected intravenously, the smell of garlic can be smelled later. However, if the nasal airflow is blocked, this sense of smell will not occur. The intravenous olfactory test proved that the injected Vitamin B1 odoranted reach to the olfactory mucosa through expired air from the lung. it still cannot produce olfaction without the involvement of flow^3–6^. Lee Sela and Noam Sobel^7^ have suggested that the bilateral cerebral cortex innervates olfaction.However, if patients with nasal congestion are caused by poor anatomical structures on one side of the nasal cavity, there is going to have a big difference in the results of bilateral nasal independent olfactory tests. It is further confirmed that olfaction is related to flow in the olfactory cleft.However, the anatomical structure is complex because the olfactory cleft is a narrow irregular cleft at the top of the nasal cavity. It is difficult to reproduce the actual isometric ratio, making it challenging to determine the flow characteristics across the olfactory cleft. Therefore, early on, Kelly, Prasad, and Wexler^8^ developed various models of the entire nasal cavity to discuss its effect on olfaction using nasal flow characteristics instead of the flow through the olfactory cleft. At present, many studies are still limited to the effect of nasal flow variation on olfaction. There is a lack of studies on the relationship between flow characteristics of the olfactory cleft and olfactory function.

The computational fluid dynamics (CFD) is a biomechanical method that has been rapidly developed in recent years to study nasal flow^9–12^. The entire nasal flow field has been studied using the CFD methods by Croce, Kimi and Kai Zhao et al.^13^. Martonen^14^ found that the deposition of odor particles increased with the increase in flow and flow velocity in the same CFD methods. There was no clear correlation between the flowrate and weight of particles in the flow. Alam et al.^15^ used CFD to analyze the change of nasal flow field and its effect on olfaction after virtual middle turbinate resection. They found that nasal resistance decreased and olfactory flux increased after middle turbinectomy, suggesting that individual anatomical differences in the olfactory cleft region and changes in flow parameters may be related to the olfactory function.

In order to investigate the correlation between the flow characteristics of the olfactory cleft and the olfactory function, this study was conducted to model the olfactory cleft of the nasal cavity in three dimensions based on CT imaging data of sinuses. The air flow, velocity, pressure, and air flow ratio in the olfactory cleft area were obtained by the hydrodynamics analysis method. The correlation between the flow parameters flowing through different positions in the olfactory cleft area and olfactory function was studied through the correlation analysis.

## Materials and methods

### Study subjects

Thirty-two eligible healthy participants were enrolled, including 16 males and 16 females. The age was 26–60 years old, with an average of (30 ± 7.08) years old. All healthy volunteers underwent clinical otolaryngology specialty examination, sinus spiral CT examination, nasal endoscopy, acoustic rhinometry and four-phase rhinomanometry, and Sniffin’ Sticks olfactory test. Inclusion criteria were: 1. a visual analogue scale (VAS) score of 0 for nasal symptoms; 2. a LUND-Mackay score of 0 for sinus CT; 3. a TDI score of > 30 for the Sniffin’ Sticks test; 4. no previous history of the chronic nasal disease and history of coronavirus infection; 5. no previous history of head trauma; 6. no history of nasal medication within the past three months. The volunteers who do not meet any one of the appeal inclusion criteria are not included in the study subjects.

This study had approval from Clinical Medical Research Ethics Committee of the Third Affiliated Hospital of Sun Yat-sen University (ZhongDaFuSanYiLun[2020] 02-079-01). Written informed consent was collected from all participating subjects. All experiments were performed in accordance with relevant guidelines and regulations.

### Three-dimensional modeling of the nasal olfactory cleft area

The CT scans of sinuses were performed at the Third Affiliated Hospital of Sun Yat-sen University on 32 enrolled participants using a Multilayer Spiral CT Scanner by Philips Healthcare with a layer spacing of 0.5 mm. Digital Imaging and Communications in Medicine (DICOM) format files were obtained. The pixel points were extracted using Materialise's Interactive Medical image Control System (MIMICS) software to create 3D point cloud data in the 3D view. The point cloud data was exported to IGES format and imported into reverse engineering software for smooth processing of the point cloud data. A surface model of the nasal cavity and sinus with biometric features was established by combining the features of biological characteristics. Finally, the 3D model of the nasal cavity, sinus, and the model of olfactory cleft area was created using Boolean operations.

### Mesh generation of nasal cavity model

Mesh generation of the nasal model is an essential step in performing hydrodynamic analysis. The quality of mesh generation directly affects the accuracy of calculation results. In this study, The Integrated Computer Engineering and Manufacturing code for Computational Fluid Dynamics (ICEM-CFD) software was used for mesh generation. The 3D model was divided into three parts: inlet, outlet, and wall. The final number of divided mesh nodes was 418,069, the number of cells was 2,473,513, there are 2,246,637 tetrahedral meshes and 171,881 triangular meshes. The final nasal cavity model with nearly 2.5 million meshes was obtained to ensure the accuracy of the calculation results.

### Setting of boundary conditions

The boundary condition of the nasal cavity was set according to the actual situation of breathing. The boundary condition of the outlet (lower end of the nasopharynx) was set as the flow boundary condition with a size of 360 ml/s; the inlet is a pressure inlet condition with a free boundary and one standard atmospheric pressure. The wall surface was a slip-free wall surface with a roughness set to 0.5 mm^16^.

### Flow field characteristic analysis

This study simulated the flow fields in the nasal cavity and paranasal sinus based on the ANSYS Workbench platform^17^. The nasal flow was considered to be an incompressible Newtonian fluid. The controlling equations of the nasal breathing process were the continuity equation and the Navier–Stokes (N–S) equation. In the simulation, the standard K-ɛ turbulence model was chosen. The fluid medium was the air with a density of 1.25 kg/m^3^ and a viscosity of 1.7894 × 10^−5^ Ns/m^2^. Fluent was used as the solver to simulate the nasal fluid dynamics and obtain the flow parameters at any intercept point of the nasal sinuses.

### Establishment of the cross-sectional system

**Figure 1:**
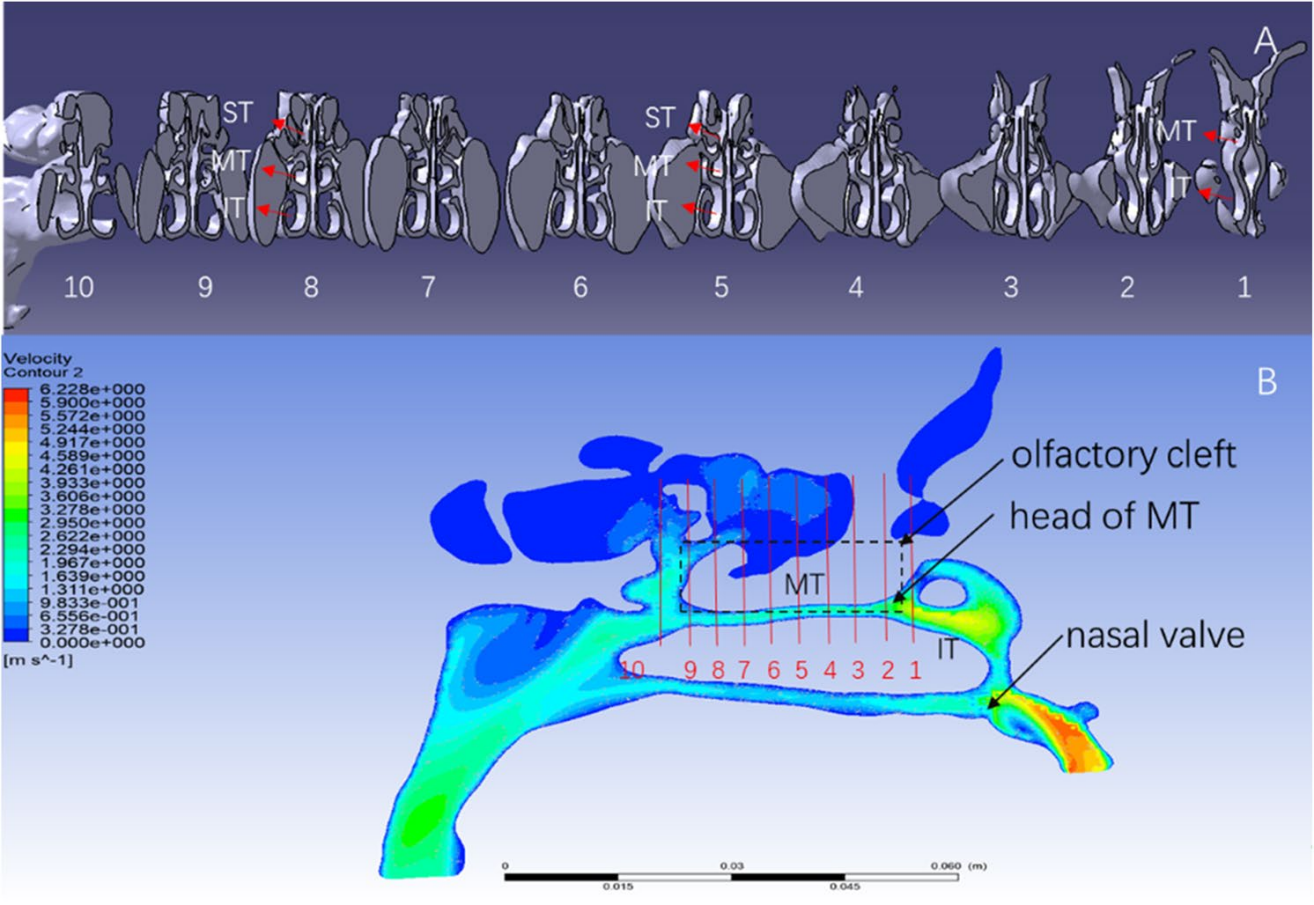
Overall sagittal segmentation view of the cross-sectional system. (**A**) Sagittal section view; (**B**) cross-sectional airflow system division (1–10 represents 10 cross-sectional systems; IT: inferior turbinate, MT: middle turbinate, ST: superior turbinate). (created by ANSYS 17.0: Academic Teaching Mechanical and CFD, ANSYS END USER CERTIFICATE, Sun Yat-sen University(#1016974)).

**Figure 2:**
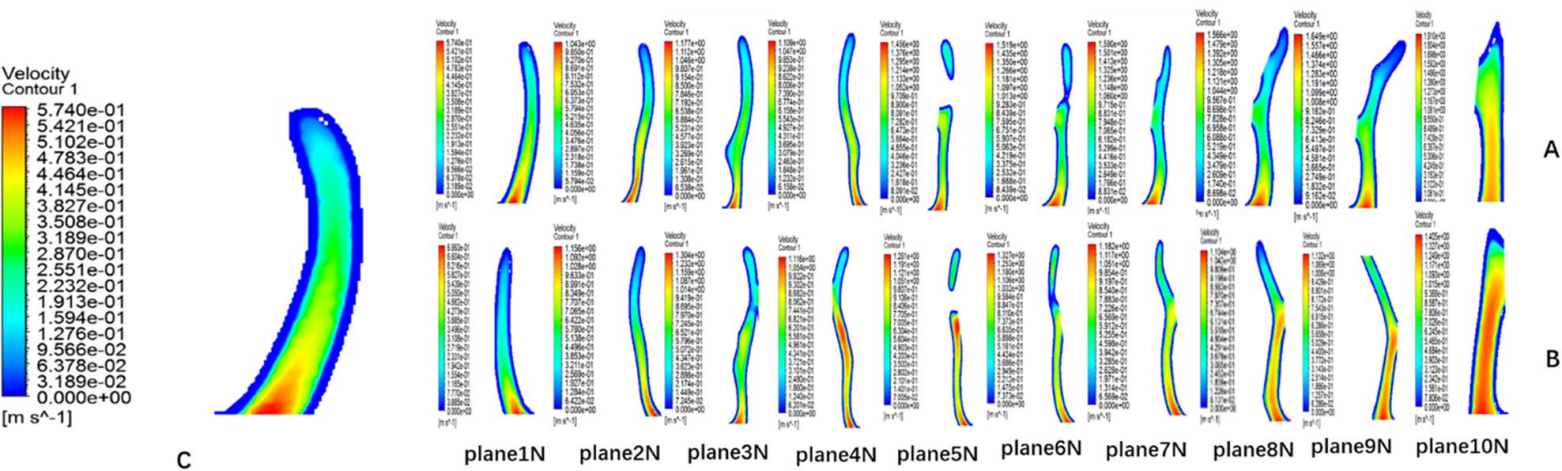
Airflow velocity diagram in the olfactory cleft area (plane1N- plane10N represent ten cross-sectional systems). (**A**) The airflow velocity of the right olfactory cleft; (**B**) The airflow velocity of the left side of the olfactory cleft.c:the airflow velocity of the initial plane of the left olfactory fissure. (created by ANSYS 17.0: Academic Teaching Mechanical and CFD, ANSYS END USER CERTIFICATE, Sun Yat-sen University(#1016974)).

To reflect the effect of the characteristics of flow parameters at different positions in the olfactory cleft area on the production of olfaction, a cross-sectional system (plane 1–10 N) was established in this study. The narrow and irregular olfactory cleft region was composed of the medial surface of the middle turbinate and the superior turbinate, and the corresponding nasal septum was divided into ten equal parts along the coronal direction (See Fig. 1). The interface data was extracted to analyze the entire olfactory cleft at different positions by using the sectioning function of software. The air flow, velocity, pressure, and air flow ratio of ten cross- sections representing other positions of the olfactory cleft area in each nasal cavity and air flow ratio data were obtained. The airflow velocity of ten cross-sections in the olfactory cleft area of volunteer No. 1 is shown in Fig. 2.

Using the method described above, all 32 participants with 64 nasal cavities were modeled in the olfactory cleft region of the nasal cavity based on the sinus CT scan data. Then, the flow flowing through the nasal olfactory cleft region was analyzed against a pre-designed cross-sectional system. The average velocity, pressure, and air flow ratio of the flow over ten cross-sections of the nasal cavity on each side of each participant and the ratio of flow through the olfactory cleft region were obtained. In each case, the nasal olfactory cleft area was divided according to the cross-sectional system.

### Sniffin’ Sticks test

Sniffin’ Sticks from Burghart, Germany, were used for the tests. The tests were conducted in a well-ventilated, quiet room. Subjects were seated and did not eat any food or drink other than water, chew gum, or smoke for at least 15 min prior to the test.

All 32 volunteers were subjected to independent olfactory testing of the bilateral nasal cavities and the left and right nasal cavities separately by the Sniffin’ Sticks test method^18^. The examiner wore cotton gloves during the examination and changed them after each test. During the test, the odor-releasing end of the sniffing pen was placed approximately 2 cm in front of the test nostrils and 2 cm in front of the nasal columella for bilateral nostril testing. The examinee was asked to sniff for only 5 s on each sniffing pen, with an interval of approximately 30 s.

During the sniffing process, the odor-releasing end of the pen did not touch the subject's skin. The unilateral nostril test was performed by closing the other nostril with a medical cotton ball without changing the shape of the nostril.

### Statistical analyses

For the statistical analyses, the SPSS 25.0 was used to statistically analyzed all data. The measurement data were expressed by $\overline{x} \pm s$. The Mann–Whitney U test was used to identify the difference of the left and right nasal cavity respectively. We also use Pearson correlation coefficient to analyze the correlation between two continuous variables, r < 0.3 means lower correlation, 0.3 < r < 0.7 means medium correlation, r > 0.7 means higher correlation. A *P*-value of < 0.05 was considered statistically significant.

## Results

### Independence test of left and right nasal olfaction and airflow parameters test results

To verify whether the left and right nasal data were independent, all 64 enrolled nasal cavities were tested separately and independently using bilateral nasal olfaction for odor threshold, odor discrimination, and odor discrimination ability. The olfactory test forms were completed and summarized as TDI scores and counted separately. Meanwhile, to compare differences between the left and right-side variables, the Mann–Whitney U test was used to verify the differences in olfactory and flow field parameters between the two sides. The results showed that there were no significant differences between the left and right-side variables. The results of the independent tests of the left and right nasal cavities could be used as independent variables to analyze the subsequent experiments, and the results are shown in Table 1.

**Table 1:** The variance analysis of variables on both sides of subjects.

Variables/results	Mann–Whitney U	Wilcoxon W	Z	*P*
TDI	403.500	931.500	− 1.461	0.144
T	483.500	1011.500	− 0.385	0.700
D	511.500	1039.500	− 0.007	0.994
I	375.500	903.500	− 1.929	0.054
Mean air flow, ml/s	445.000	973.000	− 0.900	0.368
Mean air flow pressure, Pa	510.000	1038.000	− 0.027	0.979
Mean air flow velocity, m/s	496.000	1024.000	− 0.215	0.830
Mean air flow ratio, %	440.500	968.500	− 0.960	0.337

The mean air flow, the average volume of air flow passing through a certain position of the nasal cavity; the mean air flow pressure, the average pressure of air flow passing through a certain position of the nasal cavity; the mean air flow velocity, the average velocity of air flow passing through a certain position of the nasal cavity; the mean air flow ratio, the mean ratio of the air flow through a certain section of the nasal olfactory cleft to the total air flow through the nasal cavity.

### Analysis of flow parameters in the olfactory cleft cross-sections

**Figure 3:**
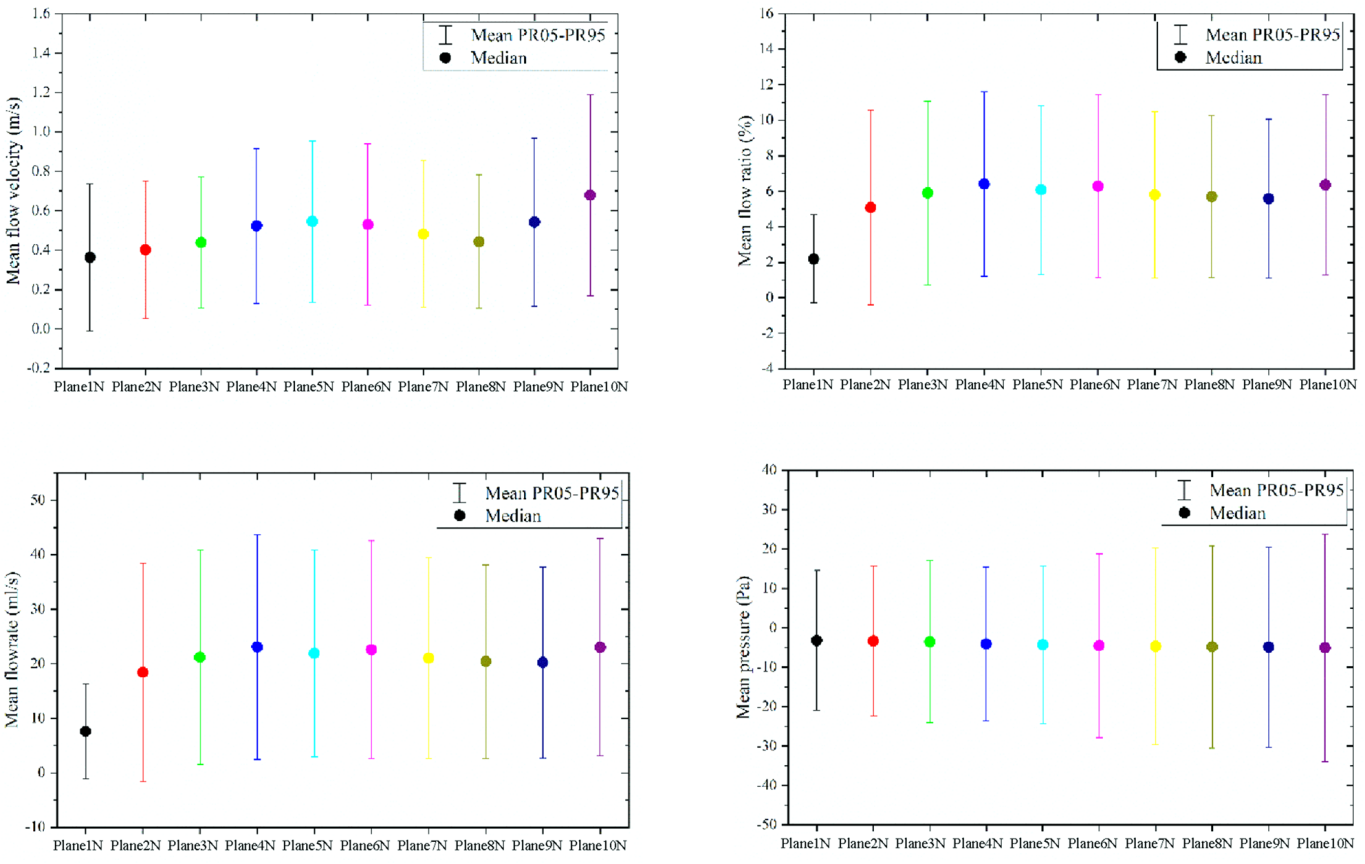
Range of statistical values of fluid characteristic parameters in the olfactory cleft region (created by Matlab R2021a, https://software.sysu.edu.cn/matlabhome).

**Figure 4:**
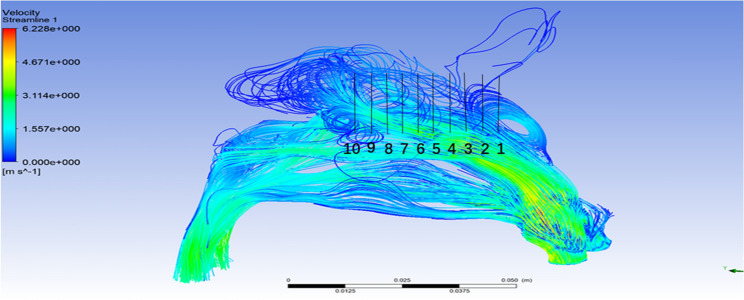
Airflow streamline in sagittal position(created by ANSYS 17.0: Academic Teaching Mechanical and CFD, ANSYS END USER CERTIFICATE, Sun Yat-sen University(#1016974)).

In order to understand the trend of airflow parameters at different positions in the olfactory cleft, the CFD analysis method was applied to model the nasal cavity and sinuses. While the olfactory cleft was divided into 10 cross-sectional positions, we can obtain the flow parameters of each cross-sectional position in olfactory region. The percentile method was used to determine the p5-p95 range of airflow parameters, and the median was used to express the mean data feature for all flow parameters of the same cross-sections from different nasal cavities.The comparative analysis showed that the four airflow parameters at the position of plane3N-plane8N in the flow through the cross-sectional system were more stable. It was also shown that the flow parameters on plane1N-plane3N were more unstable. (see Figs. 3 and 4). The anatomical positions corresponding to plane3N-plane8N in the olfactory cleft started from the junction of the anterior 1/3 and the posterior 2/3 of the middle turbinate to the posterior end of the superior turbinate. This posterior olfactory cleft region is consistent with the main distribution area of the olfactory mucosa.

### Correlation analysis of olfactory function and flow parameters in olfactory cleft region

A total of 64 cases of the airflow parameters through the nasal olfactory cleft areas were analyzed by the CFD, and the measurement data was expressed as $\overline{x} \pm s$. The mean air flow of the airflow in the olfactory cleft zone of the nasal cavity was obtained as 19.22 ± 9.74 ml/s; the mean flow velocity was 0.51 ± 0.21 m/s; the mean air flow ratio was 5.45 ± 2.52%, and the mean pressure was − 13.35 ± 6.74 Pa.

**Figure 5:**
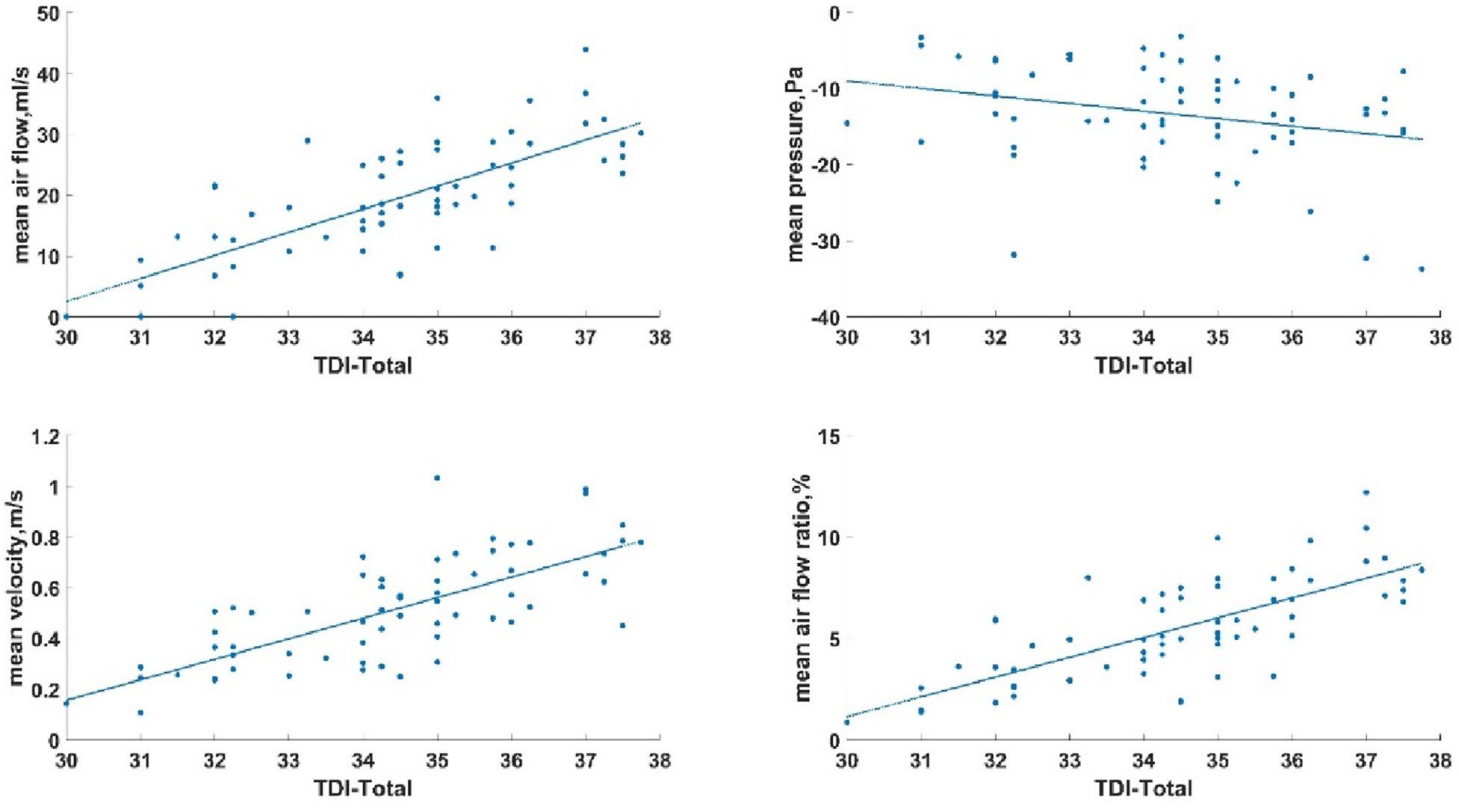
The correlation between airflow parameters and TDI score(created by Matlab R2021a, https://software.sysu.edu.cn/matlabhome).

The mean values of flow parameters of each nasal olfactory cleft area on each side of each volunteer measured by the CFD method and the values of each score representing the olfactionof each side of the nasal cavity were applied as two continuous variables. The Pearson's correlation coefficient was used to test their correlation. The statistical results showed that the mean flow (r = 0.74; *P* < 0.001), mean velocity (r = 0.72; *P* < 0.001), and air flow ratio (r = 0.73; *P* < 0.001) of the objective parameters of the olfactory cleft were highly positively correlated with olfaction. Mean pressure was not correlated with olfaction (r = 0.28; *P* = 0.026) and had a slight negative correlation with olfactory discrimination ability (r = -0.31; *P* = 0.14). The results are shown in Table 2 and Fig. 5.

**Table 2:** Results of the correlation of flow parameters in the olfactory cleft areas with the olfactory scores.

Parameters	TDI score		OT score		OD score		OI score	
	*r*	*P*	*r*	*P*	*r*	*P*	*r*	*P*
Mean air flow, ml/s	0.74	< 0.001	0.51	< 0.001	0.57	< 0.001	0.34	0.006
Mean air flow pressure, Pa	− 0.28	0.026	− 0.04	0.731	− 0.31	0.014	− 0.19	0.128
Mean air flowvelocity, m/s	0.72	< 0.001	0.41	< 0.001	0.64	< 0.001	0.34	0.005
Mean air flow ratio, %	0.73	< 0.001	0.51	< 0.001	0.52	< 0.001	0.38	0.002

Correlation analysis of the mean values of flow parameters representing each of the ten positions in the olfactory cleft areas with the measured olfactory score showed that the mean flow on plane1N-plane2N of the cross-section system correlated poorly with olfaction (r = − 0.51, r = − 0.53), while on plane4N-plane10N correlated significantly and positively with olfaction, with plane4N having the strongest correlation. The mean velocity correlated relatively poorly with olfaction on plane1N-plane3N. The correlation with olfaction gradually increased between plane4N-plane10N with plane10N being the strongest correlation overall. The correlation analysis between air flow ratio and olfaction showed the most remarkable correlation on plane4N-plane5N. On the other hand, although overall not considered having a reasonable correlation with the olfactory score, the mean pressure still has a degree of correlation in terms of the total score. Furthermore, only the breakthroughs on plane9-plane10N were mildly correlated.

## Discussion

A large amount of the current literature has mainly focused on changes in overall nasal flow on olfaction^18–21^. The olfactory mucosa in the nasal cavity is distributed in the olfactory cleft, but the relationship between flow characteristics in the olfactory cleft and olfaction has been rarely reported. We investigated the correlation between airflow parameters and olfactory function of the olfactory cleft in healthy adults. We found that three parameters, airflow velocity, air flow, and air flow ratio, were highly positively correlated with olfactory function, with the strongest correlation between air flow through the olfactory cleft and olfactory function (see Table 2). The gas flow percentage data in the olfactory cleft zone were consistent with the studies of Alam et al.^15^, The airflow reaching the olfactory area accounts for 5% to 10% of the total nasal flow. The results of our study on the effect of flow through the olfactory cleft on olfaction were consistent with the overall trend of Cherobin^19^ and Nomura^20^ in their studies on the effect of overall nasal flow on olfactory function. The olfactory function is improved when the poor anatomy of the nasal cavity is corrected and the flow of air through the olfactory cleft increases.This result could well explain the conductive olfactory dysfunction due to poor nasal anatomical structure. The reason for this may be that the differences in the anatomical structure of the nasal cavity led to changes in the flowrate and velocity of flow through the olfactory cleft region, which affects the intensity of flow stimulation of the olfactory mucosa epithelium and the deposition of olfaction particles in the flow, ultimately affecting olfactory function. This suggests that the flow parameters in the olfactory cleft could be an index of reference in the preliminary assessment of olfactory ability on the basis of the exclusion of central neurological olfactory disorders.

In the analysis of the flow characteristics at different positions of the olfactory cleft (see Figs. 3 and 4), we found that the flow parameters on plane1N-plane3N fluctuated more. At the same time, those on plane3N-plane10N were more stable, i.e., the threshold flow in the olfactory cleft area corresponding to the anterior 1/3 of the middle turbinate varied more. In the analysis of the correlation between the proportion of flow in the olfactory cleft area and olfaction, it was found that the plane4N-plane5N positions had the best correlation with olfaction, which was located at the beginning of the posterior olfactory cleft area, i.e., the junction of the anterior 1/3 of the middle turbinate. It may be because the anterior aspect of the middle turbinate acts as throttle limiting effect on the flow into the threshold of the posterior olfactory cleft region, which maintains the flow into the posterior threshold of the olfactory cleft within a stable range and thus has a more significant impact on the olfactory function. In their studies on the effect of middle turbinate resection on olfaction, Alam et al.^15^, Sicard et al.^17^ concluded that middle turbinate resection increased nasal flow and contributed to olfaction improvement. In the present study, however, we found that the anterior middle turbinate might play an essential role in maintaining the flow stability in the olfactory cleft region and that the resection of the anterior middle turbinate, although increasing the flow into the olfactory cleft, might affect the stability of flow in the posterior part of the olfactory cleft and have an adverse effect on olfactory function.

This study investigated the correlation between different positions of the olfactory cleft and olfaction in a cross-section system. Correlation analysis of the mean values of flow parameters representing each of the ten positions in the olfactory cleft areas with the measured olfactory score shown( see Table 3) that the mean air flow on plane1N-plane2N of the cross-section system correlated poorly with olfaction (r = − 0.51, r = − 0.53), while on plane3N-plane10N correlated significantly and positively with olfaction(r > 0.6), with plane4N having the strongest correlation. The mean velocity correlated relatively poorly with olfaction on plane1N-plane3N. The correlation with olfaction gradually increased between plane4N-plane10N with plane10N being the strongest correlation overall. The correlation analysis between air flow ratio and olfaction showed the most remarkable correlation on plane4N-plane5N. On the other hand, although overall not considered having a reasonable correlation with the olfactory score, the mean pressure still has a degree of correlation in terms of the total score. Furthermore, only the breakthroughs on plane9-plane10N were mildly correlated. Thus it was found that the flow parameters in the posterior region of the olfactory cleft were more correlated with olfactory function. The flow in this anatomical position was more stable and more informative for the assessment of olfaction. This area coincides with the main distribution of the olfactory zone mucosa, further validating the positive correlation between flow parameters in the olfactory cleft and olfactory function and the correlation with the stimulation of the olfactory zone mucosa by flow.

**Table 3:** Results of the correlation analysis of flow parameters in ten positions with the TDI score.

Parameters	Mean air flow		Mean pressure		Mean velocity		Mean air flow	
	*r*	*P*	*r*	*P*	*r*	*P*	*r*	*P*
Plane1N	0.50	< 0.001	− 0.19	0.125	0.48	< 0.001	0.46	< 0.001
Plane2N	0.53	< 0.001	− 0.23	0.071	0.45	< 0.001	0.49	< 0.001
Plane3N	0.63	< 0.001	− 0.14	0.257	0.56	< 0.001	0.60	< 0.001
plane4N	0.71	< 0.001	− 0.26	0.036	0.66	< 0.001	0.69	< 0.001
Plane5N	0.67	< 0.001	− 0.29	0.020	0.64	< 0.001	0.67	< 0.001
Plane6N	0.63	< 0.001	− 0.27	0.033	0.61	< 0.001	0.62	< 0.001
Plane7N	0.66	< 0.001	− 0.27	0.032	0.60	< 0.001	0.64	< 0.001
Plane8N	0.67	< 0.001	− 0.28	0.024	0.68	< 0.001	0.68	< 0.001
Plane9N	0.66	< 0.001	− 0.31	0.012	0.67	< 0.001	0.65	< 0.001
Plane10N	0.65	< 0.001	− 0.33	0.007	0.73	< 0.001	0.65	< 0.001

## Conclusions

In this study, We investigated the correlation between airflow parameters and olfactory function of the olfactory cleft in healthy adults by the CFD method. We found that three parameters, airflow, airflow velocity, and airflow ratio, were highly positively correlated with olfactory function. And the flow parameters in the posterior olfactory cleft area were more stable. Due to the limited number of research cases and the fact that olfaction is a complex psychological and physiological response with many pairs of brain nerves, it is not determined by a single factor. Therefore, the follow-up study will adopt a multicenter study and increase the number of cases to consider the synergistic effects of olfactory mucosa status and olfactory center differences on olfactory function, not only to support the quantitative assessment of conductive olfactory dysfunction. This study can be used for the evaluation of olfactory function in patients with conductive olfactory dysfunction. It can help to realize the initial assessment of olfactory function by modeling patients' CT imaging data and predicting olfaction recovery after surgery in patients with poor nasal anatomy.

## Data Availability

All data, models, and code generated or used during the study appear in the submitted article.
